# Embedding graphs in Lorentzian spacetime

**DOI:** 10.1371/journal.pone.0187301

**Published:** 2017-11-06

**Authors:** James R. Clough, Tim S. Evans

**Affiliations:** Centre for Complexity Science, Imperial College London, London, United Kingdom; University of Bristol, UNITED KINGDOM

## Abstract

Geometric approaches to network analysis combine simply defined models with great descriptive power. In this work we provide a method for embedding directed acyclic graphs (DAG) into Minkowski spacetime using Multidimensional scaling (MDS). First we generalise the classical MDS algorithm, defined only for metrics with a Riemannian signature, to manifolds of any metric signature. We then use this general method to develop an algorithm which exploits the causal structure of a DAG to assign space and time coordinates in a Minkowski spacetime to each vertex. As in the causal set approach to quantum gravity, causal connections in the discrete graph correspond to timelike separation in the continuous spacetime. The method is demonstrated by calculating embeddings for simple models of causal sets and random DAGs, as well as real citation networks. We find that the citation networks we test yield significantly more accurate embeddings that random DAGs of the same size. Finally we suggest a number of applications in citation analysis such as paper recommendation, identifying missing citations and fitting citation models to data using this geometric approach.

## Introduction

Network science seeks to understand the organisation and dynamics of complex systems by considering the structure of the interactions of their constituent parts. Studying the structure of these complex networks is a vital part of a wide variety of academic fields such as neuroscience, social science and economics [1]. Capturing aspects of a complex system as a graph can bring physical insights and predictive power. Yet these graphs can still be very complicated. Network Geometry is a developing approach in network science [2] which further abstracts the system by modelling the nodes of the network as points in a geometric space. Examples of this approach include latent space models [3], and links made between geometry and network clustering [4] and community structure [5]. In some cases this geometric embedding corresponds to physical space, such as when modelling wireless networks as random geometric graphs (RGG) [6], or considering networks embedding in geographic space [7, 8]. In other cases, the embedding geometry is not the familiar Euclidean one. Recent work discussing hyperbolic space [9–11] has revealed that non-Euclidean geometries can capture relevant network characteristics, a theme we will build upon in this paper. Scale free degree distributions naturally arise when networks are embedded in hyperbolic space [12] and there has been significant interest in embedding networks in spaces with uniform curvature [13, 14]. Local measures of curvature have also been used when characterising graph structure, for example using Ricci curvature to describe graph structure [15–18] or for routing in networks [19]. Conversely, many common network models such as Erdos-Renyi graphs and the Small-World model have been shown to lack hyperbolicity [20, 21].

As well as considering geometric models which have properties similar to real networks, it is also possible to take a given network and embed it in a geometric space by assigning coordinates to each node such that nearby nodes are more likely to share an edge than those far from each other. In a good embedding most of the network’s edges can be predicted from the coordinates of the nodes. Specifying coordinates for *N* nodes in *D* dimensions requires much less information that an *N* × *N* adjacency matrix, providing *D* ≪ *N* and so a good geometric embedding provides a concise description of a network’s structure. For example, in [22] protein interaction networks are embedded in low-dimension Euclidean space.

Notably though, in all approaches so far the target space, whether curved or not is Riemannian meaning that all distances are positive and a distance of zero exists only between a point and itself. In this paper we will discuss embedding graphs in pseudo-Riemannian geometries in which these restrictions are relaxed. Our focus here is Lorentzian geometry, which is of special importance in physics as it describes the geometry of spacetime. The simplest example is Minkowski spacetime, which is isotropic and flat, and so analogous to Euclidean space in this regard.

The graphs which are most appropriately associated with Lorentzian geometry are Directed Acyclic Graphs (DAG). This is because Lorentzian geometry has causal structure which is also present in this particular class of networks [23–25]. In the causal set approach to quantum gravity, DAGs are used as a discrete way of describing the structure of our universe’s spacetime [26]. In DAGs where the edges represent dependencies or causal relations, it is natural to embed in a spacetime because in physics it is separation in the universe’s Lorentzian spacetime which determines whether one event can causally affect another or not. The acyclic property of DAGs allows their nodes to be ordered, with all edges respecting that order and in our approach, this natural ordering of nodes corresponds to the time direction in the embedding spacetime.

The approach we outline here seeks to match the causal structure of a DAG (the ‘ancestors’ and ‘descendants’ of each vertex) with the underlying causal structure of Minkowski spacetime. For this reason, this approach is not applicable to undirected graphs (where there is no edge direction to describe the direction of a causal relationship) or to directed graphs with cycles (as closed causal loops cannot exist in Minkowski spacetime).

In this paper we will show how to find reasonable spacetime coordinates for each node in a DAG so that the causal relationships in the network are well matched with the causal relationships in the embedding spacetime. The process has two steps: firstly, spacelike and timelike separations are estimated for each pair of nodes using tools from the causal set approach to quantum gravity. Secondly, a generalised form of Multidimensional Scaling (MDS) is used to find spacetime coordinates which best respect these separations. For clarity we begin by reviewing MDS and generalising it to pseudo-Riemannian spaces, then discuss estimating separations on the graph.

## Methods

### Review of classical MDS

Suppose we have *N* objects, which live in a *D* dimensional Euclidean space, and we are given the squared Euclidean distance, *S*_*ij*_ between each pair *i* and *j*. We wish to find the coordinates of the objects, which will be *D* dimensional vectors, **x**_*i*_ for each object *i*, such that they fit the constraint that |**x**_*i*_ − **x**_*j*_|^2^ = *S*_*ij*_. The classical MDS algorithm solves this problem by using this *N* × *N* matrix of square distances, S, and then constructing the double centred matrix $B=-\frac{1}{2}JSJ$ where $J=\text{1}-\frac{1}{N}1.1^{T}$. It can then be shown (see [27]) that

$$\begin{array}{c} B=X^{T}X \end{array}$$ (1)

where X is an *N* × *D* matrix of co-ordinate vectors **x** which satisfy the constraint of recovering the original distances, and with the centre of mass of the coordinates at the origin. As well as being real and symmetric, B is also guaranteed to be semi-positive definite, i.e. it has no negative eigenvalues. So we can then find (up to a factor of a rotation) the coordinates in X by decomposing B into

$$\begin{array}{c} B=U^{T}\Sigma U \end{array}$$ (2)

where **Σ** is a diagonal matrix of the eigenvalues of B, and U a matrix of its eigenvectors. A solution is given by

$$\begin{array}{c} X=\sqrt{\Sigma }U \end{array}$$ (3)

This process yields coordinates in *N* dimensions, but only *D* of the eigenvalues will be non-zero. It is possible retrieve coordinates in fewer dimensions, by using only the largest $\hat{D}$ eigenvalues and their corresponding eigenvectors. The larger eigenvalues correspond to principle components, meaning that using them as the coordinates minimises the square difference between the original distances we started with, and those calculated from these inferred coordinates. These coordinates are in this sense the most accurate $\hat{D}$ dimensional representation of the original data and it is in this manner that MDS can be used for dimensionality reduction.

As well as this simple version of the algorithm, faster approximations also exist. Landmark MDS [28] is a two step process, where first a small number of ‘landmark’ points have their positions fixed to each other using the usual MDS method, and second, the remaining points are fixed using only their distances to the landmarks. Pivot MDS [29] provides further improvements by iteratively updating the positions of the landmarks, or pivots, using the rest of the points, and then updating the positions of the rest of the points using the pivots. Although we omit the details here these faster methods are also applicable to the approach we describe below and we include implementations of them in our code which is freely available [30].

### Lorentzian multidimensional scaling

Minkowski spacetime is a combination of a *d*-dimensional Euclidean space, and one time dimension forming a (*d* + 1) dimensional spacetime. A point *i* in this spacetime, has coordinates **x**_*i*_ consisting of a time coordinate, $x_{i}^{0}$, and spatial coordinates $x_{i}^{k}$, with *k* = 1, 2, …, *d*. The Minkowski separation between two such spacetime points *i* and *j* is given by

$$\begin{array}{c} M_{ij}=M\left(x_{i},x_{j}\right)=-c^{2}{\left(x_{i}^{0}-x_{j}^{0}\right)}^{2}+\sum_{k=1}^{d}{\left(x_{i}^{k}-x_{j}^{k}\right)}^{2} \end{array}$$ (4)

where *c* is the speed of information flow. We may always choose to work in terms of coordinates where this speed is equal to 1. For instance in special relativity, *c* is the speed of light but we may measure distance in light-seconds, and time in seconds such that the numerical value of *c* is 1 in these units. In Minkowski spacetime, pairs of points, *i* and *j* can then be classified into three types: for a *M*_*ij*_ > 0 the pair is spacelike separated, for a *M*_*ij*_ < 0 the pair is timelike separated, while pairs on the boundary, or light-cone, defined by *M*_*ij*_ = 0 are called lightlike separated. In physics, timelike separated events can be causally connected, meaning that information can travel the past event to the future event. Spacelike separated points cannot be causally related because they are separated by too much space and too little time for any signal to reach from one to the other.

We can now ask the same question that classical Euclidean MDS poses: given pairwise separations *M*_*ij*_, for points in this spacetime, can we recover coordinates which respect these separations?

Proceeding with the classical Euclidean algorithm we can construct the double centred matrix B as before using $B=-\frac{1}{2}JMJ$. However we now encounter a problem when decomposing B. Previously **Σ**, the eigenvalues of B, were guaranteed to be non-negative, but now we find one negative eigenvalue corresponding to the time dimension’s negative sign in Eq 4. Since we need to take the square root of these eigenvalues, and we want real number coordinates, this is a problem.

It turns out that the changes required to the classical MDS algorithm are remarkably simple (details are given in S1 Appendix)). Instead of looking for a matrix of coordinates X such that $B=X^{\text{T}}X$, we now search for solutions to

$$\begin{array}{c} B=X^{T}GX \end{array}$$ (5)

where G is matrix representing the metric of the embedding space. For classical MDS with its Euclidean space G is just the identity matrix so this factor drops out from the analysis. Instead we now choose G to represent the Minkowski metric, which in our conventions is a diagonal matrix with −1 in the first column and +1 in the others. Since B is still real and symmetric, we decompose B into the matrix of eigenvectors U and the diagonal matrix of eigenvalues **Σ** as before but we now need solutions to

$$\begin{array}{c} X^{T}GX=U^{T}\Sigma U\;. \end{array}$$ (6)

The difference to classical MDS is that we associate each negative eigenvalue in **Σ** with a corresponding entry of −1 in the in the diagonal metric matrix G. Positive eigenvalues are linked to entries of +1 in G as before, while zero eigenvalues correspond to zeros in G. The solution we seek is therefore $X=U\sqrt{\left|\Sigma \right|}$ as the negative signs of **Σ** are captured by G allowing us to take the square root of |**Σ**| to leave us with real coordinates in X, mimicking the procedure followed in classical MDS. For our Minkowski space example, the coordinates derived from the eigenvector with the largest negative eigenvalue are labelled the time coordinates, and those derived from the eigenvectors of the *d* largest positive eigenvalues are the *d* spatial coordinates.

### Graph distance in spacetime

In the Euclidean case, the geometric approach says that nodes that are near to each other should be more likely to share an edge than those which are far away. In the simplest case, a random geometric graph, nodes are placed randomly in a Euclidean space and pairs of nodes within some threshold distance share an edge while remaining pairs do not [31–33]. This means that the distance in the Euclidean space between two nodes can be approximated in the network by the number of edges on the shortest path between them [22].

What is a useful approach to take in for DAGs in Lorentzian geometry? In many networks which form DAGs the reason that the graph is acyclic is that the edges represent some kind of causal relationship. This is the case for citation networks, family trees and scheduling problems. In physics the possibility of causal relationships is determined by geometry. One event can cause another only if they are timelike separated (*M*_*ij*_ < 0 in Eq (4)), and cannot if they are spacelike separated (*M*_*ij*_ > 0). Therefore we seek to assign coordinates to the nodes such that those pairs connected by some directed path are timelike separated, while the remaining unconnected pairs are spacelike separated. To be precise, since timelike separation is a transitive relationship but the edges in the network might not be, we are trying to achieve this criteria on the transitive closure of the network [34].

This is the same construction as in the causal set approach to quantum gravity. In this theory the underlying structure of the universe’s spacetime is a *causal set*, which is a locally finite partially ordered set. Causal sets form transitively closed DAGs and so whether or not the real spacetime of the universe actually does consist of a causal set, the theory does give us a natural way of relating a discrete graph to points in a continuous spacetime. Although we use results from the causal set literature, we do not discuss them in detail and direct the reader to [24, 26, 35, 36] for more details.

### Estimating geodesic distance

Given a DAG, how can we estimate the separation between each pair, *M*_*ij*_ using only the graph structure? To do this we use a simple model of uniformly scattered points in Minkowski spacetime. We are effectively trying to fit a DAG to a uniformly scattered causal set model, in the same way that approaches embedding networks in Euclidean space using MDS are fitting the network to a random geometric graph.

Let $G_{D}\left(N\right)$, which we will call a causal set graph, be a DAG constructed as follows. Each of the *N* vertices is associated with a coordinate in a *D* dimensional Minkowski spacetime, chosen uniformly at random from [0, 1]^*D*^. This range of coordinates is chosen in our examples for simplicity but this does not need to be the case in general. A directed edge is then placed between any distinct pair of vertices whose coordinates are timelike separated with the edge direction from past to future.

It was conjectured by Myrheim [37] and later shown in [38, 39] that for timelike separated vertices *i* and *j* in $G_{D}\left(N\right)$ the squared length of *longest path*, respecting the direction of the edges is proportional to their timelike separation, in the limit of *N* → ∞. We will therefore use the squared length of the longest directed path to estimate of *M*_*ij*_ for timelike separated pairs.

**Fig 1:**
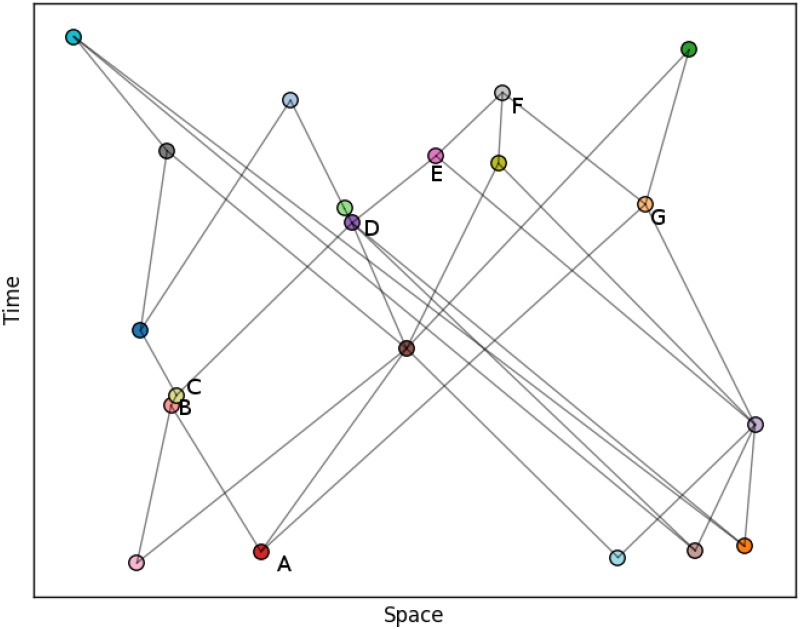
Estimating Minkowski separations in a graph. The Minkowski separation between nodes A and F, *M*_*AF*_ is approximated as −25 units as 5 is the number of edges in the longest direction-respecting path between them. Nodes B and G are spacelike separated. To estimate this separation we find a pair of points in their mutual past and future. In this case, the only such pair is (A, F). The naive spatial separation between (B, G) is then given by the timelike separation between (A, F) so is +25 units. Note, only the edges not implied by transitivity have been drawn.

Finding the distance between spacelike pairs is more challenging and there is currently no method which is both as accurate and as easily calculated as the longest path is for timelike pairs [24]. Approximations are known, and we will use a very simple one, described in [40, 41] as ‘naive spatial distance’. Suppose we have two disconnected vertices *i* and *j* in $G_{D}$ meaning they are spacelike separated. We then look for all pairs of nodes, *k* and *l*, where *k* is in the future of both *i* and *j* while *l* is both their pasts. We then choose the pair, k* and l*, with the minimum longest path amongst all the pairs. The timelike separation between k* and l* is then used as an estimate for the spacelike separation between *i* and *j*. If no such pair exists, we set the spacelike distance of *i* and *j* equal to some maximal distance which is a parameter of the algorithm. In the examples shown here, we used the length of the longest path in the graph as this parameter. Fig 1 gives an example.

This estimate is simple and at first appealing, although it becomes increasingly inaccurate in more than two dimensions when *N* → ∞ (hence ‘naive’). Nonetheless we find it is sufficient for our purposes. We tried using the two-link method described in [41] but found that even for graphs with 2000 nodes too many cases had no two-links and so the spacelike separation couldn’t be calculated resulting in a worse embedding. This is partly because it is inaccurate only for large graphs but also because in the MDS algorithm each point’s coordinates is fixed by many separations, both timelike and spacelike which limits the effect of noise from a few poor estimations.

Given a graph, these timelike and spacelike separation estimates define our separation matrix M (where timelike separation has the − sign in our conventions), on which we can perform the Lorentzian MDS algorithm described above to embed it in Minkowski spacetime, as summarized in the algorithm box below.

**DAG embedding algorithm**

1. For every pair *i* and *j* connected by a directed path, find the length of the longest directed path between them, *L*_*ij*_.
2. For every other pair, find the naive spacelike distance *N*_*ij*_.
3. Create separation matrix, M, such that $M_{ij}$ is $-L_{ij}^{2}$ if there is a path from *i* to *j* and $N_{ij}^{2}$ otherwise.
4. Use Lorentzian MDS with M as the input matrix of squared separations.

## Results

Once the Lorentzian MDS algorithm has estimated coordinates in *D*-dimensional Minkowski spacetime for each vertex in the graph, how can we assess the accuracy of the embedding? Informally, a good embedding is one where the edges, or chains of edges in the graph correspond to timelike separation of the nodes and non-edges correspond to spacelike separation. Perhaps surprisingly, not every DAG can be embedded perfectly so that this relation is respected for all pairs [42], even for an arbitrarily large dimension of spacetime. Even where a DAG can be perfectly embedded the discrete structure of the graph will introduce noise meaning that we will not necessarily recover the original coordinates exactly.

To quantify the effectiveness of an embedding we will take the estimated coordinates and use them to rebuild the graph by again placing edges only between timelike separated pairs. If there are edges between the same vertices in the recreated graph and in the original graph, the embedding is an accurate one, and if not then it is poor. As in [22] we will measure this using the sensitivity (the fraction of the correct edges which were predicted) and specificity (the fraction of correct non-edges which were predicted). We are effectively considering the estimated coordinates as a method of predicting edges in the original graph.

**Fig 2:**
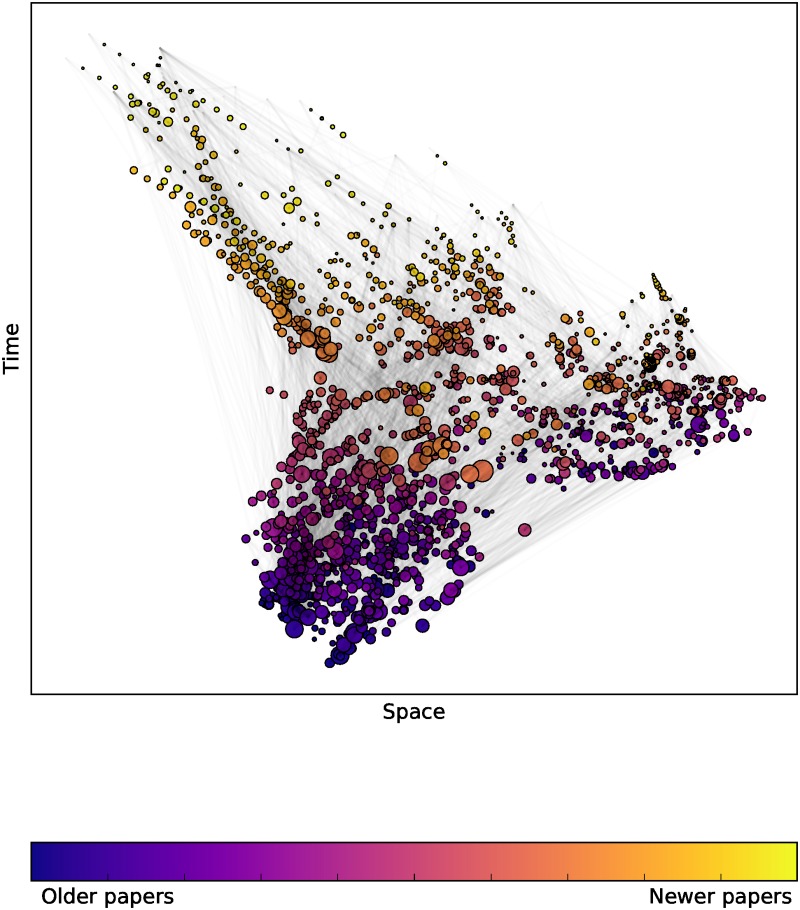
Papers from arXiv embedded in Minkowski space using Lorentzian MDS. A visualisation of a *D* = 1 + 1 embedding of the top 2000 most cited papers in the hep-ph citation network, where node size is proportional to the number of citations. Node colour corresponds to publication date, and in both cases this correlates strongly with the time coordinate obtained from the embedding algorithm. The hep-ph citation network appears more broad in space indicating more pairs of papers which are spacelike separated from each other.

**Fig 3:**
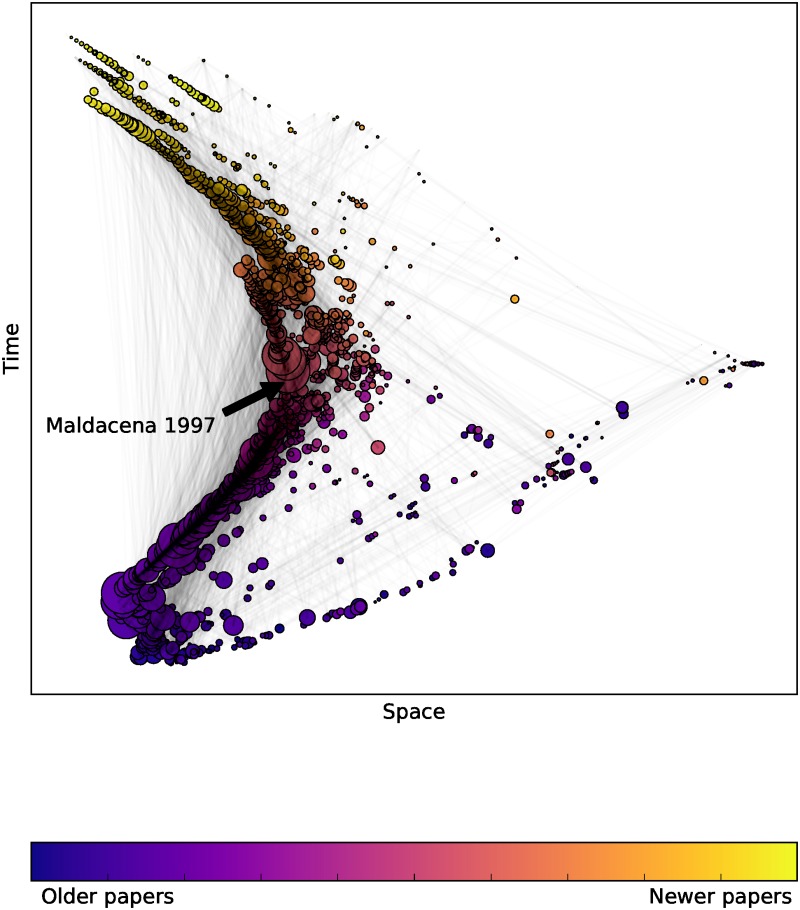
Papers from arXiv embedded in Minkowski space using Lorentzian MDS. A visualisation of a *D* = 1 + 1 embedding of the top 2000 most cited papers in the hep-th citation network, where node size is proportional to the number of citations. Node colour corresponds to publication date, and in both cases this correlates strongly with the time coordinate obtained from the embedding algorithm. In contrast to the hep-ph network, the hep-th citation network has most of its papers in a long chain indicating more timelike separated pairs. We highlight the central placement of the most cited paper in that citation network hep-th/9711200, Maldacena’s paper “The Large N Limit of Superconformal Field Theories and Supergravity”. The visually ‘narrow’ citation network of hep-th and ‘broad’ hep-ph agrees with our previous findings in [25].

We will illustrate our method on several citation networks: the citations within the hep-th section (high energy physics theory) of arXiv up to 2003 produced for the 2003 KDD cup [43], similarly for the hep-ph section (high energy physics phenomenology) [43], for the Supreme Court of the US (SCOTUS) [44], Minkowski spacetime causal set graphs as described above, and finally random DAGs. By a random DAG we mean an Erdős-Rényi random graph, with the nodes placed in a random order and edges directed with respect to that order, and finally the graph is transitively completed. Figs 2 and 3 give examples of a *D* = 2 embedding for the hep-ph and hep-th citation networks from the KDD cup dataset.

**Fig 4:**
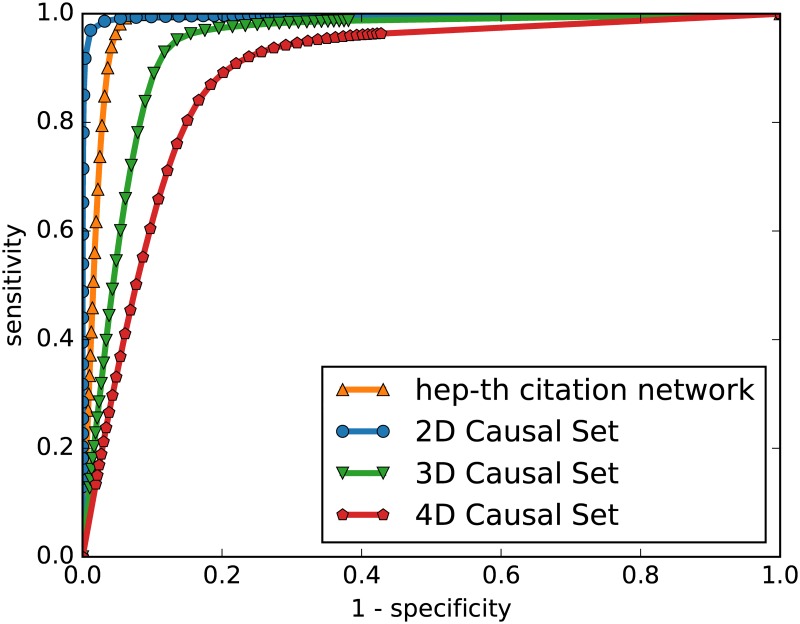
Sensitivity of results. Curves showing the sensitivity and specificity of embeddings into *D* = 2 Minkowski space of causal set graphs in two-, three- and four-dimensional spacetimes, and of a citation network from the hep-th section of arXiv, all with *N* = 1000 vertices.

To compare the sensitivities and specificities of the various embeddings we use the established method of the area under the receiver-operator curve (AUC). Varying a continuous parameter, the sensitivity and specificity of the embedding is measured, and plotted, as in Fig 4. and the area under this curve describes the embedding’s quality.

The continuous parameter we will vary is the speed of light (or the speed information can be transferred) in the embedding Minkowski space, *c*. Previously, we have set *c* = 1, but varying this speed will change which nodes are connected in new network generated from the MDS coordinates. Now, nodes *i* and *j* are connected if their coordinates satisfy

$$\begin{array}{c} -c{\left(x_{i}^{0}-x_{j}^{0}\right)}^{2}+\sum_{k=1}^{d}{\left(x_{i}^{k}-x_{j}^{k}\right)}^{2}<0 \end{array}$$ (7)

For small values of *c*, very few nodes are connected and so the specificity is high (few false positives) but the sensitivity is low (many false negatives). For large values of *c*, many nodes are connected and so the reverse is true.

**Fig 5:**
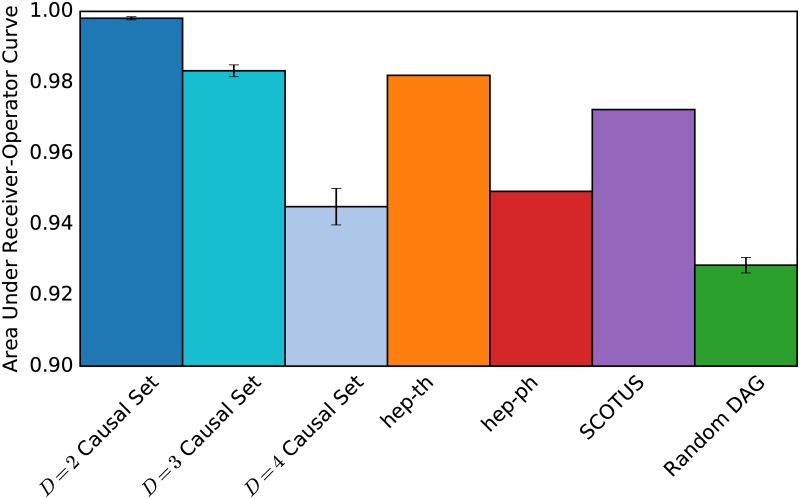
The quality of embeddings. Area under the curve (AUC) values represent the quality of an embedding. Here we show the AUC values for embedding graphs with 2000 nodes into *D* = 2 Minkowski spacetime. A value of 1 represents a perfect embedding, and a value of 0.5 is random chance. The two-dimensional causal set graph has, as expected, the highest value, since there must be coordinates allowing a perfect embedding (the original coordinates used when building that graph). Higher dimensional causal sets can be embedded less well, but still better than a random DAG (far right). Error bars show the standard deviations of this measurement over 20 randomly generated examples. Notably, the three citation networks we use as examples have significantly higher values that the random DAG illustrating that they have structure which allows a better fit to Minkowski spacetime. The comparatively better fit of the hep-th network over the hep-ph network into 2-dimensional spacetime agrees with our result in [25].

We will compare various DAGs of size *N* = 1000, and results are shown in Fig 5. The causal set graphs $G_{2}\left(1000\right)$ are stochastically generated as described previously. The random DAGs are Erdos-Renyi graphs with the edges then directed according to a random ordering on the nodes, and then transitively completed [45]. The number of edges in the original ER graph is chosen to roughly match the KDD Cup citation networks but the comparatively poor AUC scores of the random DAGs is a robust to changes in this parameter.

## Discussion and conclusions

We foresee this approach being applied in two cases. Firstly, there are applications where the network data of interest is in the form of a DAG and so as is the case for causal sets the natural geometric approach to take is a Lorentzian one. Finding an effective geometric embedding of a network provides a powerful tool for the analysis of that network as it allows standard geometric techniques and intuition to be used. Calculations of network properties can be made more efficient, for example, when finding optimal routes from one node to another, the node coordinates provide local information which can improve routing algorithms [10]. Models built on hyperbolic spaces can yield scale free, clustered networks with community structure illustrating the remarkable power that geometric approaches have to recover complex network properties [12], and we suggest the same could be true of DAGs in Lorentzian spacetimes.

A second application is in dimensionality reduction. Our method may be used where there is some domain specific reason to think that the appropriate target space should be Lorentzian, because datapoints are associated with points in time and are related in some causal way. Generalising the equations for classical MDS allows it to be used on any metric signature, even though we have focused only on the Lorentzian signature here. To our knowledge this pseudo-Riemannian output is a new development, although some manifold learning techniques exist which can take pseudo-Riemannian manifolds as their input [46, 47]. We note that when performing the embedding one can find multiple negative eigenvalues, suggesting that embedding in spaces with more than one timelike dimension is also possible, as are potential embeddings into Lorentzian manifolds other than Minkowski space, incorporating curvature or preferred directions. Furthermore dimensionality reduction algorithms which begin by building a graph of nearest neighbours (such as Isomap [48]) could be adapted to have a Lorentzian spacetime as the target space using the kind of longest-path approach we describe here.

The visualisation of networks is a problem in its own right, and two or three dimensional embeddings from our method can be combined with standard plotting software to give network visualisations for DAGs in which the causal ordering is explicit. Such visualisations are used in bibliometrics to help identify distinct fields or assist literature reviews [49].

When standard dimensionality reduction techniques are used on high-dimensional datasets it is common to see complicated, abstract features of the data represented by directions in the reduced coordinates which may represent underlying degrees of freedom in the mechanism generating the data. See for example in [48] where this effect is apparent on images and handwritten digits.

In the cases of citation analysis we conjecture that the spatial dimensions that result from a geometric approach correspond to similarity in the topic of a paper, and so our approach yields spatial similarities between papers while accounting for the time difference in their publication. Once estimated coordinates are known, the idea that nodes may be ‘similar’ can be expressed as nodes being close in their spatial coordinates. Two papers that do not cite each other, or share authors or citations might still be close in the embedded coordinates since these are calculated globally using information from all vertices and edges. Closeness in the embedded coordinates is then a similarity measure which can be used for applications such as clustering, paper recommendation, and centrality measures, the effectiveness of which is an avenue for future work.

Another use of this approach is where edges in a network are placed primarily according to some geometric rule but their connections are also governed by some smaller second order effect. It may only be possible to measure the smaller effect once we have accounted for the primary geometric one by assigning coordinates. We can see this phenomenon clearly when the geometric embedding is in real geographic space, such as in [50] where accounting for geographic distance in phone-call data allows more accurate prediction of the second order effect of shared language.

The focus in this paper has been to show how the MDS approach may be adapted to embed networks in a spaces with a distinct time direction, such as Minkowski spacetime and other non-Riemannian spaces. The algorithms we used provide an illustration but we expect more and scalable algorithms will be possible with further work. In our implementation the majority of the computation time is taken up calculating the spacelike distances in the graph. Since this involves counting many longest paths it is more challenging than the calculations of effective distances in traditional MDS methods and we expect improvements on our simple approach should be possible. The second part of the process is the assignment of coordinates given the spacetime distances. A central result here is that working with a spacetime is a straightforward adaptation of the standard Euclidean MDS methods. This means that the established MDS algorithms can be applied in this context so we expect that existing techniques, e.g. Landmark MDS [28] or Pivot MDS [29], will deliver optimal performance.

In conclusion our approach is a general one that we hope finds use in a wide range of applications in network embedding, visualisation, geometric analysis and dimensionality reduction problems where a Lorentzian target space may be appropriate. In particular datasets of causally related events or objects naturally correspond to an embedding in Lorentzian spacetime and so are obvious candidates for this approach. To this end we direct readers to [30] where a Python implementation of our approach is available, along with examples including those used to generate some of the diagrams in this paper.

## Supporting information

S1 Appendix Derivation of Lorentzian MDS. (TEX)
